# Entanglement and quantum correlation measures for quantum multipartite mixed states

**DOI:** 10.1038/s41598-023-29438-7

**Published:** 2023-02-17

**Authors:** Arthur Vesperini, Ghofrane Bel-Hadj-Aissa, Roberto Franzosi

**Affiliations:** 1 DSFTA, University of Siena, Via Roma 56, Siena, 53100 Italy; 2 INFN Sezione di Perugia, Perugia, 06123 Italy; 3 QSTAR & CNR - Istituto Nazionale di Ottica, Largo Enrico Fermi 2, Firenze, 50125 Italy

**Keywords:** Quantum information, Qubits, Theoretical physics

## Abstract

Entanglement, and quantum correlation, are precious resources for quantum technologies implementation based on quantum information science, such as quantum communication, quantum computing, and quantum interferometry. Nevertheless, to our best knowledge, a directly or numerically computable measure for the entanglement of multipartite mixed states is still lacking. In this work, (i) we derive a measure of the degree of quantum correlation for mixed multipartite states. The latter possesses a closed-form expression valid in the general case unlike, to our best knowledge, all other known measures of quantum correlation. (ii) We further propose an entanglement measure, derived from this quantum correlation measure using a novel regularization procedure for the density matrix. Therefore, a comparison of the proposed measures, of quantum correlation and entanglement, allows one to distinguish between quantum correlation detached from entanglement and the one induced by entanglement and, hence, to identify separable but non-classical states. We have tested our quantum correlation and entanglement measures, on states well-known in literature: a general Bell diagonal state and the Werner states, which are easily tractable with our regularization procedure, and we have verified the accordance between our measures and the expected results for these states. Finally, we validate the two measures in two cases of multipartite states. The first is a generalization to three qubits of the Werner state, the second is a one-parameter three qubits mixed state interpolating between a bi-separable state and a genuine multipartite state, passing through a fully separable state.

## Introduction

Entanglement has assumed an important role in quantum information theory and in the development of the quantum technologies. It is considered as a valuable resource in quantum cryptography, in quantum computation, in teleportation and in quantum metrology^1^. Nevertheless, entanglement remains elusive and the problem of its quantification in the case of a general system, is still open^2,3^. In the last decades, several approaches have been developed to quantify the entanglement in the variety of states of the quantum realm. However, the rigorous achievements in the explicit quantification of entanglement, are limited to bipartite systems case^4^. Entropy of entanglement is uniquely accepted as measure of entanglement for pure states of bipartite systems^5^, while entanglement of formation^6^, entanglement distillation^7–9^ and relative entropy of entanglement^10^ are largely acknowledged as faithful measures for bipartite mixed systems^11^. An extensive literature is devoted to the study of entanglement in multipartite systems. Over time, different approaches have been proposed including, e.g. in the case of pure states, the study of the equivalence classes in the set of multipartite entangled states^12,13^, whereas, the study of entanglement in mixed multipartite states have been addressed, e.g., with a Schmidt measure^14^ or with a generalisation of concurrence^15,16^. In the last years, have been proposed entanglement estimation-oriented approaches and derived from a statistical distance^17^ concept, as, for instace, the quantum Fisher information^18–21^.

In the case of pure states, entanglement and correlation are completely equivalent, therefore an appropriate measure of quantum correlation can provide also an entanglement measure. On the contrary, in the case of mixed states, one can observe states that manifest correlations detached from entanglement^11,22^.

In the present work, (i) we propose a new directly computable measure of quantum correlation for mixed states. Moreover, (ii) we propose a numerically computable entanglement measure for mixed states, which is derived from the quantum correlation measure through a regularization process. In the following we first derive the quantum correlation measure for mixed states and, from the latter, we derive an entanglement measure valid for the same class of states. Finally, we report four examples of the application of quantum correlation and entanglement measures. We have considered two well-known classes of states: a general Bell diagonal state and the Werner states. In addition, we have applied the quantum correlation and entanglement measures to Werner state generalization to three qubits, and to a one-parameter three qubits mixed states interpolating between a bi-separable state and a genuine multipartite state, passing through a fully separable state.

## Entanglement distance for mixed states

###  Quantum correlation distance

We consider the Hilbert space ${{\fancyscript {H}}} = {{\fancyscript {H}}}^{0} \otimes {\fancyscript {H}}^{1} \cdots {{\fancyscript {H}}}^{M-1}$ tensor product of *M* two qubits Hilbert spaces. The Hilbert–Schmidt distance *D* between two general square matrices, *A* and *B*, is given by

1 $$\begin{aligned} D(A,B) = \sqrt{\dfrac{1}{2} \textrm{tr}[(A-B)^\dagger (A-B)] } . \end{aligned}$$

We derive from the latter, the distance between two close density matrices of a quantum state in ${{\fancyscript {H,}}}$ by

2 $$\begin{aligned} d^2_{_{dm}} (\rho ,\rho +d\rho )= \dfrac{1}{2} \textrm{tr}[(d\rho )^\dagger (d\rho )]. \end{aligned}$$

The Hilbert–Schmidt distance is not the only possible choice, e.g. the Bures’ distance represents an appropriate alternative option. The infinitesimal variation $d\rho$ of state $\rho$ is

3 $$\begin{aligned} d\rho&= \sum ^{M-1}_{j=0} d{\tilde{U}}^\mu \rho + \rho \sum ^{M-1}_{\mu =0} d{\tilde{U}}^{\mu \dagger } \nonumber \\&= -i \sum ^{M-1}_{\mu = 0} \sum ^3_{j=1} \left[ \sigma ^\mu _j {\rho } \right] n^\mu _j d \xi ^\mu , \end{aligned}$$

where

4 $$\begin{aligned} d{\tilde{U}}^\mu = -i ( {\varvec{\sigma }}_\textbf{n})^\mu d \xi ^\mu \end{aligned}$$

and with [,], we mean the commutator. Here and in the following we use the notation $({\varvec{\sigma }}_\textbf{n})^\mu = (\textbf{n}^\mu \cdot {\varvec{\sigma }}^\mu )$, and for $\mu =0,\ldots ,M-1$, we denote by $\sigma ^\mu _1$, $\sigma ^\mu _2$ and $\sigma ^\mu _3$ the three Pauli matrices operating on the $\mu$-th qubit, where the index $\mu$ labels the spins. We have

5 $$\begin{aligned} d^2_{_{dm}} (\rho ,\rho +d\rho )= \sum ^{M-1}_{\mu ,\nu =0}g_{\mu \nu } (\rho , \textbf{n}) d\xi ^\mu d\xi ^\nu , \end{aligned}$$

where

6 $$\begin{aligned} \!\!\!\!\!\! g_{\mu \nu } (\rho , \textbf{n}) = \dfrac{1}{2} \sum ^3_{i,j=1} \textrm{tr}[\rho \{ \sigma ^\mu _i, \sigma ^\nu _j \} \rho -2 \rho \sigma ^\mu _i \rho \sigma ^\nu _j ] n^\mu _i n^\nu _j , \end{aligned}$$

with {,} we mean the anticommutator. We define the quantum correlation for the state $\rho$ as

7 $$\begin{aligned} C(\rho ) =\inf _{ \{\textbf{n}^\nu \}_\nu } \textrm{tr}(g(\rho , \textbf{n}) ) . \end{aligned}$$

Since $C(\rho )$ derives from a distance, we name it quantum correlation distance (QCD). Furthermore, the quantum correlation is the inferior value of the trace of *g* when the unit vectors are varied, therefore its numerical value is invariant under local unitary transformations. We have

8 $$\begin{aligned} \sum ^{M-1}_{\mu =0} g_{\mu \mu }(\rho , {\textbf{n}}) = M {\textrm{tr}}(\rho ^2)-\sum ^{M-1}_{\mu =0} \sum ^3_{i,j=1} {\textrm{tr}}[\rho \sigma ^\mu _i \rho \sigma ^\mu _j ] n^\mu _i n^\mu _j . \end{aligned}$$

Finally, by defining the matrices $A^\mu (\rho )$, for $\mu =0,\ldots ,M-1$, whose entries are

9 $$\begin{aligned} A^\mu _{ij}(\rho ) = \textrm{tr}[\rho \sigma ^\mu _i \rho \sigma ^\mu _j ] , \end{aligned}$$

we obtain the closed-form expression for the QCD of $\rho$,

10 $$\begin{aligned} C(\rho ) =\sum ^{M-1}_{\mu =0}\Big (\textrm{tr}(\rho ^2) - \lambda ^\mu _{max} (\rho ) \Big ) = \sum ^{M-1}_{\mu =0}C_\mu (\rho ) \end{aligned}$$

where, for $\mu =0,\ldots ,M-1$, $\lambda ^\mu _{max} (\rho )$ is the maximum of the eigenvalues of $A^\mu (\rho )$, and $C_\mu (\rho ) = \textrm{tr}(\rho ^2) - \lambda ^\mu _{max} (\rho )$ is the QCD of the subsystem $\mu$. The QCD is a directly computable measure of the degree of correlation of $\rho$. Remarkably, Eq. (10) contains two competing terms. The first term is named Purity, which takes account of the degree of statistical mixing of $\rho$, its upper bound 1 corresponds to a pure state. The second term ranges between 0 and 1 and derives from the degree of correlation of $\rho$, with the lower value, 0, corresponding to the higher correlation.

The time complexity of the obtained formula for the QCD is that of $D\times D$ matrix multiplications, that is $o(D^3)$, where *D* is the dimension of the full Hilbert space. In particular, the QCD possesses a closed formula and do not require any optimisation (other than finding the largest eigenvalue of $3\times 3$ matrices). This is in contrast with other measures of quantum correlation which, to our best knowledge, all require time-costly optimisation procedures, except for some specific classes of states^11^.

The QCD (10) fulfills the following requirements for a *bona fide* measure of quantum correlation^11^:

1. $C_\mu (\rho )=0$ if $\rho \in {\fancyscript {C}}_\mu$, i.e. if $\rho$ is classical in the subsystem $\mu$. Indeed, $\forall \rho \in {\fancyscript {C}}_\mu$ we can write $\rho = \sum _j p_j\rho _j^{\mu _C} \otimes |j\rangle \langle j|^\mu$, where the $\{|j\rangle ^\mu \}$ form an orthonormal basis in ${\fancyscript {H}}^\mu$, $\mu _C$ is the complement of subsystem $\mu$ and $\sum _j p_j=1$. Then $\exists {\varvec{n}}^\mu$ such that $\forall j,\, ^\mu\langle j |{\varvec{n}}^\mu \cdot {\varvec{\sigma }}^\mu |j\rangle ^\mu =\pm 1$, hence $\lambda ^\mu _{max} (\rho )=1$ and $C_\mu (\rho )=0$. It results $C(\rho )=0$ if $\rho \in {\fancyscript {C}}$, i.e. if $\rho$ is fully classical.
2. $C(U\rho U^\dagger )=C(\rho )$, i.e. it is invariant under local unitary transformations.
3. In the case of a pure state $\rho ={|{\psi }\rangle }{\langle {\psi }|}$,$C({|{\psi }\rangle }{\langle {\psi }|})$ reduces to the measure of entanglement valid for pure states that some of us have derived in former work^23^. This confirms that for pure states a genuine correlation measure provides also an entanglement measure.

### Entanglement distance

As stated above, for a mixed state, the existence of quantum correlation is not a sufficient condition to guarantee the presence of entanglement. To extract from a given state $\rho$ its entanglement essence, we now propose a procedure of regularization of $\rho$, repurposing our measure of quantum correlations to catch the true degree of entanglement owned by $\rho$.

Given a state $\rho$, we consider all of its possible decomposition $\{p_j,\rho _j\}$, such that

11 $$\begin{aligned} \rho = \sum _j p_j \rho _j , \end{aligned}$$

where $\sum _j p_j = 1$ and $\textrm{tr}[\rho _j] = 1$. Also, we consider all the possible local partial transformation on qubit $\mu$:

12 $$\begin{aligned} \rho _U^\mu (\{p_j,\rho _j,U_j^\mu \}) = \sum _j p_j U_j^\mu \rho _j U^{\mu \dagger }_j , \end{aligned}$$

where, for each *j*, $U_j^\mu$ is an *SU*(2) local unitary operator acting on qubit $\mu$. We define the entanglement measure for state $\rho$

13 $$\begin{aligned} E(\rho ) = \inf _{\{p_j,\rho _j\}} \Big \{ \sum _{\mu =1}^{M-1} \inf _{\{U_j^\mu \}} C_\mu \left( \rho ^\mu _U(\{p_j,\rho _j,U_j^\mu \}) \right) \Big \}. \end{aligned}$$

Since the definition $E(\rho )$ derives from a distance, we named it entanglement distance (ED). Note that, similarly to the QCD, one can define $E_\mu (\rho )$ as the ED of subsystem $\mu$, simply discarding the complement in the sum on $\mu$ in (8). The ED (13) fulfills the following requirements for a suitable measure of quantum entanglement:

i) $E_\mu (\rho )=0$ if $\rho \in {\fancyscript {S}}_\mu$, that is if $\rho$ is separable in $\mu$. Indeed, it then admits a decomposition $\{p_j,\rho _j\}$, where, for each *j*, $\rho _j= ({\mathbb {I}}^\mu + {\varvec{\sigma }}_{\textbf{n}_j}^\mu )/2 \otimes \rho _j^{\mu _C}$, where. Thus, it is always possible to determine local partial operators $U_j^\mu$, such that, after transformation (12) it results $\rho _U^\mu = \sum _j p_j|j\rangle \langle j|^\mu \otimes \rho _j^{\mu _C}$ and, from property 1), it follows our statement. It results $E(\rho )=0$ if $\rho \in {\fancyscript {S}}$, that is if $\rho$ is fully separable.
ii) Reciprocally, if $E(\rho ) =0$, then $\rho$ is separable. First of all, we note that, for each $\mu =0,\ldots ,M-1$, $\lambda ^\mu _{max} (\rho ) \le \textrm{tr}(\rho ^2)$. In fact, for each $\mu$ and for each unit vector $\textbf{n}^\mu$ it is possible to determine a unitary local operator *U*, so that ${\textrm{tr}}[\left( \rho ({\varvec{\sigma }}_{\textbf{n}})^\mu \rho ({\varvec{\sigma }}_{\textbf{n}})^\mu \right) ] = {\textrm{tr}}[{\tilde{\rho }} {\sigma }_3^\mu {\tilde{\rho }} {\sigma }_3^\mu ]$, where ${\tilde{\rho }} = U\rho U^\dagger$. Furthermore ${\textrm{tr}}[{\tilde{\rho }} {\sigma }_3^\mu {\tilde{\rho }} {\sigma }_3^\mu ] = \sum _j {\tilde{\rho }}_{jj}^2+2 \sum _{i\ne j} \pm |{\tilde{\rho }}_{ij}|^2\le \sum _j {\tilde{\rho }}_{jj}^2+2 \sum _{i\ne j} |{\tilde{\rho }}_{ij}|^2 = {\textrm{tr}}[{\tilde{\rho }}^2] = {\textrm{tr}}[\rho ^2]$. Moreover, for each pair $i\ne j$, $\exists \mu$ such that the term $|{\tilde{\rho }}_{ij}|^2$ appears in ${\textrm{tr}}[({\tilde{\rho }} {\sigma }_3^\mu )^2]$ with a negative sign. Yet, $E(\rho ) =0$ implies that there exist a decomposition of $\rho$, let’s say ${\overline{\rho }}$, for which
  14 $$\begin{aligned} \sup _{\textbf{n}^\mu } \textrm{tr}[{\overline{\rho }} ({\varvec{\sigma }}_{\textbf{n}})^\mu ) {\overline{\rho }} ({\varvec{\sigma }}_{\textbf{n}})^\mu )] =\textrm{tr}[{\overline{\rho }}^2] \end{aligned}$$
  for each $\mu$. We hence have $|{\overline{\rho }}_{ij}|^2 =0$ for each $i\ne j$. But this implies that ${\overline{\rho }}$ is diagonal and then $\rho$ separable.

For a given density matrix decomposition $\{p_j,\rho _j\}$, the minimization on the local unitary partial transformations, entailed by Eq. (13), can be addressed by studying the local minima of $C(\rho (\{p_j,\rho _j,U_j\}))$ under variation of $\{U_j\}$. Nevertheless, it can be proven that such fixed points do correspond only to cases where $E(\rho )=0$, hence to separable states. Therefore, the minima of (13) in the case of non-separable states, do not correspond to fixed points, but rather to nonlocal (boundary) minima. Remarkably, these fixed points of the minimization procedure (13) can, at least in some cases, be realized by a decomposition $\{p_j,\rho _j\}$ including entangled pure states $\rho _j$. In particular, for two-qubits states diagonal in the Bell basis (the Bell-diagonal (BD) states, see^24,25^) the fixed points can always be realized on the eigen-decomposition (hence, where the $\rho _j$ are Bell states). This of course greatly simplify the problem, as the full exploration of the $\{p_j,\rho _j\}$-space is avoided. It is worth emphasizing that BD states are representative of the larger class of two-qubits states of maximally mixed marginals (that is, for which $\forall \mu$ and $\forall j$, $\textrm{tr}[\rho \sigma _j^\mu ]=0$, see^24^), hence (13) is tractable in the same manner for this class of states. Leaning on numerical evidences, we further conjecture that, for a given state $\rho (\pmb {\gamma })$ depending on parameters $\pmb {\gamma } = (\gamma _1,\gamma _2,...)$, the decomposition realizing the minimum (13) is the same in the whole parametric domain of $\pmb {\gamma }$, and can hence be inferred from the fixed points found in the domains where this state is separable, if such a domain exists. This suggests that the minimization over all possible decompositions $\{p_j,\rho _j\}$ might in fact possess *non-trivial* general solutions, depending on the considered class of states. Here, by “non-trivial solutions” of the minimization procedure, we mean solutions which do not require to find the decomposition of $\rho$ in terms of pure product-states $\rho _j=\bigotimes _\mu ({\mathbb {I}}^\mu + ({\varvec{\sigma }}_{\textbf{n}_j})^\mu )/2$. A subsequent more thorough work on such a classification of the solutions of this procedure could thus lead to an entanglement measure of relatively low computational cost, in particular for systems symmetric under qudit permutations, and with low ${\text {rank}}(\rho )$.

## Applications

### Bell diagonal states

As a first and seminal example of application of this procedure, we consider general BD states. They can be expressed as:

15 $$\begin{aligned} \rho _{BD}(\{p_\alpha \})&= \sum _{\alpha =1}^4 p_\alpha |\psi _\alpha \rangle \langle \psi _\alpha |\nonumber \\&= \frac{1}{4}\Big ({\mathbb {I}} + \sum _i c_i \sigma _i^0\sigma _i^1 \Big ), \end{aligned}$$

where the $|\psi _\alpha \rangle$ are the four Bell states: $|\psi _\pm \rangle =\frac{1}{\sqrt{2}}(|00\rangle \pm |11\rangle )$ and $|\phi _\pm \rangle =\frac{1}{\sqrt{2}}(|01\rangle \pm |10\rangle )$. Furthermore, we have $\forall i,\,|c_i|\le 1$, and the $c_i$ are such that the vector $(c_1,c_2,c_3)$, fully characterizing the state, belongs to the tetrahedron ${\fancyscript {T}}$ of vertices $(-1,1,1),\,(1,-1,1),\,(1,1,-1),\,(-1,-1,-1)$. The separable BD states belong to the octahedron ${\fancyscript {O}}$ of vertices $(\pm 1,0,0),\,(0,\pm 1,0),\,(0,0,\pm 1)$, corresponding to the condition $\forall \alpha ,\,p_\alpha \le 2$, and the classical BD states are located on the Cartesian axis $(c_1,0,0),\,(0,c_2,0),\,(0,0,c_3)$^24,25^.

**Figure 1:**
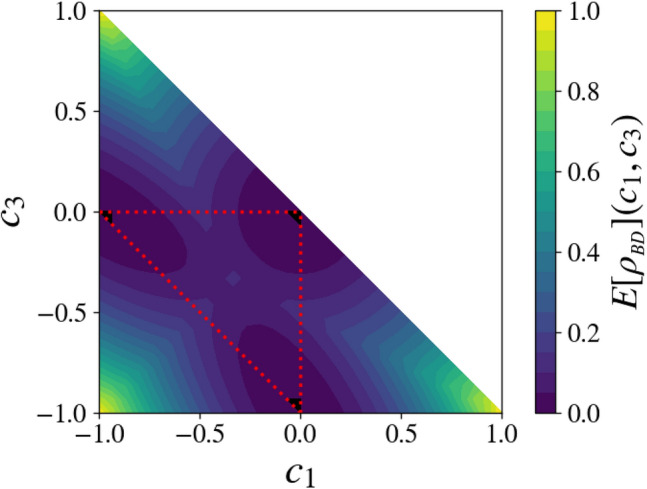
Quantum correlations $C[\rho _{BD}](c_1,c_2=c_1,c_3)/2$ for a face of the BD state tetrahedron ${\fancyscript {T}}$, corresponding to a mixture of three Bell states. The red dotted line defines the smaller triangle where the state is separable, according to the PPT criterion^26,27^. The vertices of the large triangle correspond to pure Bell states. Those of the red dotted triangle, of vanishing QCD, correspond to equal-weight mixtures of two Bell states, which are evidently the three only *classical* states in the represented domain.

**Figure 2:**
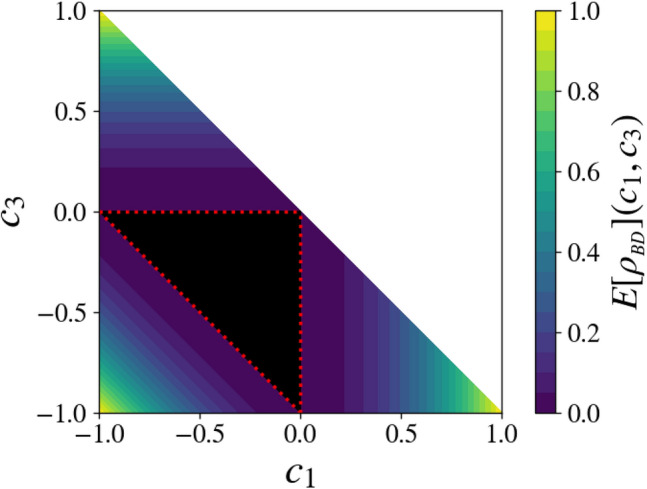
Entanglement distance $E[\rho _{BD}](c_1,c_2=c_1,c_3)/2$ for a face of the BD state tetrahedron ${\fancyscript {T}}$, corresponding to a mixture of three Bell states. The red dotted line defines the smaller triangle where the state is separable, according to the PPT criterion^26,27^ and a number of alternative derivations available in the literature (see e.g.^24^). Values below the threshold of $10^{-3}$ have been represented in black to emphasize that they correspond to a numerical zero, given the level of precision allowed by such time-costly minimization. The vertices of the large triangle correspond to pure Bell states, and those of the smaller black triangle to equal-weight mixtures of two Bell states.

Direct calculation yields the following result for the QCD of general BD states

16 $$\begin{aligned} C(\rho _{BD}(\{p_\alpha \})=2\sum _{\alpha =1}^4 p_\alpha ^2 -4\max _{P\{i,j,k,l\}}\Big \{p_ip_j +p_kp_l\Big \}, \end{aligned}$$

where the maximum is taken on all permutations $P\{i,j,k,l\}$ of the indices $\{1,2,3,4\}$. Figure 1 shows the QCD of BD states on a face of ${\fancyscript {T}}$. We were not able to find a simple analytic solution of the minimization procedure for the most general case of BD states. However, numerical minimization (for these calculations, we have applied a gradient steepest-descent method) provided us with empirical evidence that the procedure (22) also leads for these states to the squared concurrence, as shown in Fig. 2, which represent a face of the tetrahedral domain of BD states. It it interesting to note that the ED, as the concurrence and unlike the QCD, is constant on planes parallel to the boundary faces of the separability region: the ED of any given state indeed equates the QCD of the closest point located on a hinge of ${\fancyscript {T,}}$ hence the closest mixture of only two Bell states.

### Werner states

Let us now consider the two-qubit Werner states (WS)^28^, which stems as a special case of BD state, for which a simple analytical solution for the proposed procedure is available. WS are used as a testbed since they illustrate many features of mixed-states entanglement^7^. Using Eq. (15), they can simply be expressed as

17 $$\begin{aligned} \rho _{W}(p)=\rho _{BD}\big (\frac{p}{3},\frac{p}{3},\frac{p}{3},(1-p)\big ) . \end{aligned}$$

Via direct calculations, one gets for the QCD of the WS

18 $$\begin{aligned} C(\rho _W(p) ) = 2(1-\dfrac{4}{3} p)^2 . \end{aligned}$$

WS yields a relatively simple solution to the minimization procedure (22). Indeed, as it can be easily verified, if we set

19 $$\begin{aligned}&U_{|\psi _+\rangle }(\theta )=U^\mu _z(\theta )U^\mu _x(\pi ), \nonumber \\&U_{|\psi _-\rangle }(\theta )=U^\mu _z(\pi -\theta )U^\mu _x(\pi ), \text { and}\nonumber \\&U_{|\phi _+\rangle }=U_{|\phi _-\rangle }={\mathbb {I}}, \end{aligned}$$

with $\mu =0,1$ arbitrarily chosen, the fixed points are found for $\theta =\arccos {(\frac{3}{2p}-2)}$. This last expression has a solution if and only if $p\ge 1/2$, which is the parametric region of separability for $\rho _W(p)$ (as can be verified by application of the positive partial trace criterion, see^27^). Hence, $E(\rho _W)=0$ for $p\ge 1/2$. For $p<1/2$ numerical minimization yields $E(\rho _W)=4p^2 - 4p + 1$. This corresponds to $\theta =0$ uniformly on this whole domain, which is also the value previously determined at $p=1/2$: hence, the minimum after this point cease to be a fixed point, but keeps the last position in terms of the parameters governing the rotations. One can understand this as the fixed point reaching the boundary of the parametric domain as the geometry of the state is changing continuously, becoming a simple point on a slope, located at this boundary. All together, for Werner states, the result of our entanglement measure exactly equates twice the square of the concurrence^6^, that is

20 $$\begin{aligned} E(\rho _W(p) ) = 2\Theta (1/2-p)(1-2p)^2 , \end{aligned}$$

**Figure 3:**
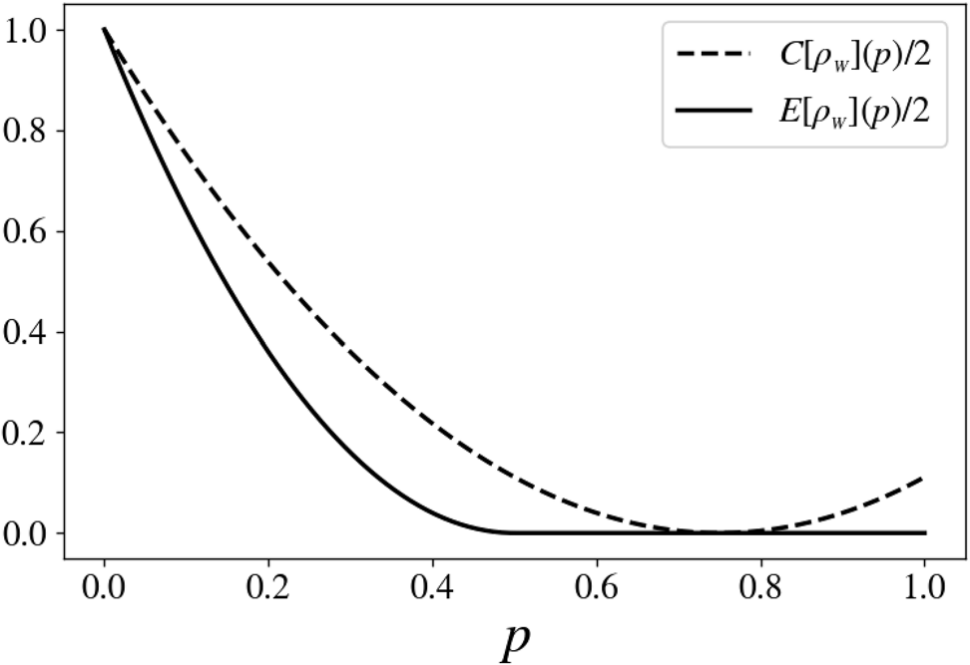
$C[\rho _W](p) /2$ and $E[\rho _W](p) /2$ versus *p* for state (17). It is clear that the state $\rho _W(p=0)$ is, as expected, the maximally-entangled, and that the states $\rho _W(p>1/2)$ are fully-separable, as can be verified using the PPT criterion^26,27^. This plot emphasizes that separable states can contain quantum correlation (i.e. not be classical). Note that, here $E[\rho _W](p) /2=C_2^2[\rho _W](p)$, that is, the ED equates twice the squared concurrence for 2-qubits Werner states.

Figure 3 shows $C(\rho _W(p) ) /2$ versus *p*, there it is clear that the only state with no quantum correlation, i.e. *classical state* according to the conventional terminology^11^, is the one corresponding to the value $p=3/4$, whereas the maximally quantum-correlated state is that of $p=0$. On the other hand, the state is entangled only in the region $p<1/2$, and separable otherwise, a well-known fact that can be easily checked by application of the positive partial transpose (PPT) criterion^26,27^. Alternatively, one can find, in the separable region, the expression of $\rho _W$ convex combination of (non-orthogonal) product states, using a more involved calculation resorting to the so-called Bloch representation.

### Generalized Werner states

Let us now consider as a multipartite example the following one-parameter density matrix

21 $$\begin{aligned} \rho _{W_3} (p) = p |GHZ_+\rangle \langle GHZ_+| + \dfrac{(1-p)}{8} {\mathbb {I}}_8 , \end{aligned}$$

where $|GHZ_+\rangle =(|000\rangle + |111\rangle )/\sqrt{2}$, ${\mathbb {I}}_8$ is the identity operator of the three-qubits Hilbert space and $0\le p\le 1$. This is a generalization of the Werner states to three qubits, termed generalized Werner states^29–31^. The states $\rho _{W_3}(p)$ are known to be fully separable for $0\le p \le 1/5$^29,30,32^ and genuinely multipartite entangled states in the region $3/7 < p \le 1$^33^. In the region $1/5 < p \le 3/7$ the states $\rho _{W_3}(p)$ are bi-separable yet inseparable under any fixed bipartition^33^. Via direct calculations, one gets

22 $$\begin{aligned} C(\rho _{W_3}(p)) = 3 p^2 . \end{aligned}$$

**Figure 4:**
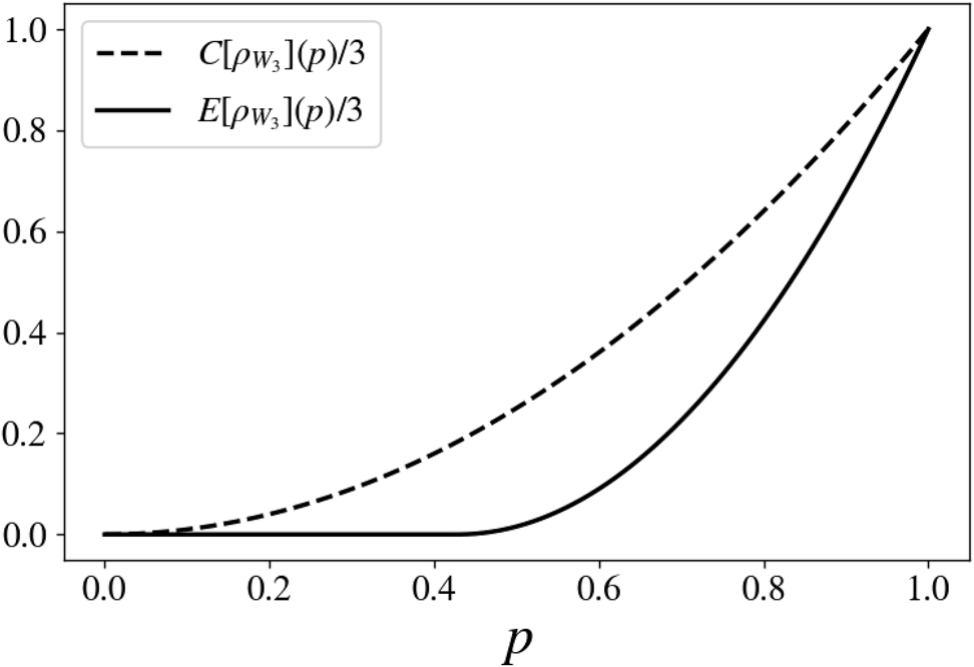
$C[\rho _{W_3}](p) /3$ (dotted line) and $E[\rho _{W_3}](p) /3$ (continuous line) versus *p* for state (21). It is clear that the state $\rho _{W_3}(p=1)$ is, as expected, the maximally entangled, and that the states $\rho _{W_3}(p > 3/7)$ are not separable. The latter are genuinely three-partite entangled states.

Numerical minimization provided the values for the ED shown in Fig. 4. There, we report in dotted line the QCD per qubit and continuous line the ED per qubit for the states $\rho _{W_3}(p)$. Figure 4 clearly shows that $ED(\rho _{W_3}(p)) > 0$ only for $p> 3/7$, that is when the states are generally entangled. As for the region $1/5 < p \le 3/7$ where ED should not be zero according to (ii), we got numerical zero which we assume corresponds to very weak, but finite values. We interpreted this as a consequence of the fact that, in this region, the states $\rho _{W_3}(p)$ are not separable under any fixed bipartition, thus assuming the decomposition of the form $\sum _j \rho ^1_j \otimes \rho ^{23}_j + \rho ^2_j \otimes \rho ^{13}_j + \rho ^3_j \otimes \rho ^{12}_j$. Hence the regularization procedure reaches easily small values for the ED.

### Three qubit states interpolating between bi-separable and genuine entangled states

Let consider a further multipartite example, that is the one-parameter density matrix

23 $$\begin{aligned} \rho _{3} (p) = w_+ |GHZ_+\rangle \langle GHZ_+ |+w_2| \psi _2\rangle \langle \psi _2| + w \dfrac{(1-p)}{8} {\mathbb {I}}_8 , \end{aligned}$$

where

24 $$\begin{aligned}{}&w_+ = p[1-4p(1-p)] , \\&w_2\ =(1- p)[1-4p(1-p)] , \\&w\ \ \,= 4p(1-p) , \end{aligned} $$

$| \psi _2\rangle = |0\rangle (|00\rangle + |11\rangle )/\sqrt{2}$ and $0\le p\le 1$. For $p=0$, $\rho _{3} (p=0)$ is a pure bi-separable state, for $p=1/2$, $\rho _{3} (p=1/2)$ is a maximally mixed state of three qubits and for $p=1$, $\rho _{3} (p=1)$ is a pure maximally entangled state. Via direct calculations, one gets

25 $$\begin{aligned} C(\rho _{3}(p)) = \dfrac{(1-2p)^4}{2} \left[ 5-10p+11p^2-(1-p) \sqrt{1-2p(1-p)} \right] . \end{aligned}$$

**Figure 5:**
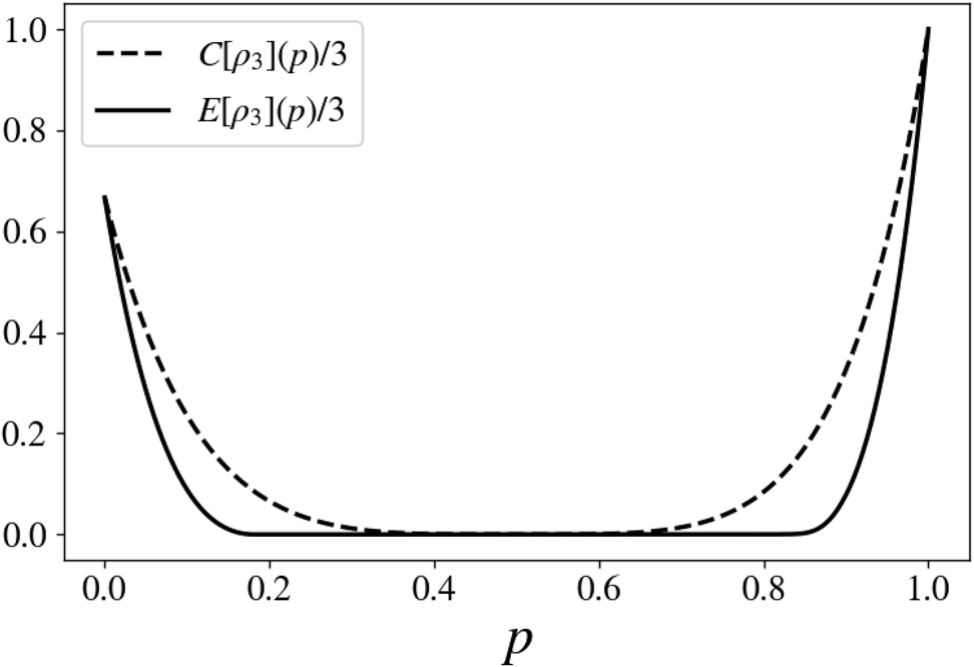
$C[\rho _3](p) /3$ (dotted line) and $E[\rho _3](p) /3$ (continuous line) versus *p* for state (23). It is clear that the state $\rho _3(p=1)$ is, as expected, the maximally entangled one, and that the states $\rho _3(p > 0.81)$ or $\rho _3(p < 0.18)$ are not separable.

Using numerical minimization, we have obtained the results for the ED shown in Fig. 5. In this figure, we report in dotted line the QCD per qubit and in continuous line the ED per qubit, for the states $\rho _{3}(p)$. Figure 5 shows that $E(\rho _3(p)) > 0$ for $0\le p \lessapprox 0.18$ and for $0.81 \lessapprox p \le 1$. Furthermore, the maximum value for ED per qubit in the region $0\le p \lessapprox 0.18$ is located at $p=0$ and has the value 2/3. 2/3 is the maximum value for ED per qubit, in the case of bi-separable three qubits states. This confirms that the states of this region are stably bi-separable and that the state $|\psi _2\rangle \langle \psi _2|$ has the maximum local degree of entanglement. The maximum value for ED per qubit in the region $0.81 \lessapprox p \le 1$ is located at $p=1$ and has value 1. Therefore, the states of this region are not separable and, at least close to $p=1$, are certainly genuinely entangled. For $0.18< p<0.81$ the entanglement is numerically null, thus suggesting the states of this region are separable or bi-separable yet inseparable under any fixed bipartition, hence not genuinely three-partite entangled states. Remarkably, the QCD is null only for the state corresponding to $p=1/2$, which is the maximally mixed one.

## Summary

The increasing interest in quantum information experimental applications, and the consequent demand for the development of skills in quantum state manipulation, has made pressing the development of effective measures of correlation and entanglement, valid for the general case of mixed multipartite states. Also, such measures are expected to be easily computable. For multipartite systems, a broad range of measures has covered pure states and mixed states, among which a Schmidt measure and a generalization of concurrence have been proposed. Nevertheless, the application of these measures to general multipartite mixed states still shows some issues. The main aim of the present work is to propose alternative measures for correlation and entanglement based on a geometric framework. Remarkably, the latter fact, makes the validity of our measures dimension independent.

Our goal in this work has been to derive (i) a directly computable and genuine quantum correlation measure and (ii) a numerically computable entanglement measure, from the geometric properties of the projective Hilbert space describing a quantum multipartite system. For mixed states, the quantum correlation is not a faithful measure of entanglement. In our derivation, to extract from a given state $\rho$ its entanglement essence, we have defined a regularization procedure for the density matrix that allows our measure of quantum correlation to catch the true degree of entanglement owned by $\rho$. The calculation of the entanglement involves a minimization procedure that, in the general case requires numeric simulations. In the latter sense, the entanglement corresponds to the inferior value of the correlation of a density matrix $\rho$ when local decompositions and local unitary transformations are operated on it. We have proved that the entanglement and quantum-correlation measures derived do satisfy the requirements for suitable measures of these quantities. To test our quantum correlation and entanglement measures, we have applied them to two classes of mixed two-qubit states of which are well-known the entanglement properties, the Bell diagonal states and the Werner states, and we have verified the accordance between our measures and the expected results. Furthermore, we have applied the quantum correlation and entanglement measures to Werner state generalization to three qubits, and to a one-parameter family of three qubits mixed states. These latter interpolate between a bi-separable state and a genuine multipartite state, passing through a fully separable state. Also in these cases of multipartite states, then we have verified a satisfactory agreement between the behaviours deduced by our measures and the ones expected or already known in the literature.

## Data Availability

The datasets used and/or analysed during the current study available from the corresponding author on reasonable request.

## References

[1] Gühne O, Toth G (2009). Entanglement detection. Phys. Rep..

[2] Sperling J, Walmsley IA (2017). Entanglement in macroscopic systems. Phys. Rev. A.

[3] Giovannetti V, Mancini S, Vitali D, Tombesi P (2003). Characterizing the entanglement of bipartite quantum systems. Phys. Rev. A.

[4] Horodecki R, Horodecki P, Horodecki M, Horodecki K (2009). Quantum entanglement. Rev. Mod. Phys..

[5] Popescu S, Rohrlich D (1997). Thermodynamics and the measure of entanglement. Phys. Rev. A.

[6] Wootters WK (1998). Entanglement of formation of an arbitrary state of two qubits. Phys. Rev. Lett..

[7] Bennett CH, DiVincenzo DP, Smolin JA, Wootters WK (1996). Mixed-state entanglement and quantum error correction. Phys. Rev. A.

[8] Bennett CH (1996). Purification of noisy entanglement and faithful teleportation via noisy channels. Phys. Rev. Lett..

[9] Horodecki M, Horodecki P, Horodecki R (1998). Mixed-state entanglement and distillation: Is there a “bound” entanglement in nature?. Phys. Rev. Lett..

[10] Vedral V, Plenio MB, Rippin MA, Knight PL (1997). Quantifying entanglement. Phys. Rev. Lett..

[11] Adesso G, Bromley TR, Cianciaruso M (2016). Measures and applications of quantum correlations. J. Phys. A: Math. Theor..

[12] Dür W, Vidal G, Cirac JI (2000). Three qubits can be entangled in two inequivalent ways. Phys. Rev. A.

[13] Briegel HJ, Raussendorf R (2001). Persistent entanglement in arrays of interacting particles. Phys. Rev. Lett..

[14] Eisert J, Briegel HJ (2001). Schmidt measure as a tool for quantifying multiparticle entanglement. Phys. Rev. A.

[15] Coffman V, Kundu J, Wootters WK (2000). Distributed entanglement. Phys. Rev. A.

[16] Carvalho ARR, Mintert F, Buchleitner A (2004). Decoherence and multipartite entanglement. Phys. Rev. Lett..

[17] Braunstein SL, Caves CM (1994). Statistical distance and the geometry of quantum states. Phys. Rev. Lett..

[18] Pezzé L, Smerzi A (2009). Entanglement, nonlinear dynamics, and the Heisenberg limit. Phys. Rev. Lett..

[19] Hyllus P (2012). Fisher information and multiparticle entanglement. Phys. Rev. A.

[20] Tóth G (2012). Multipartite entanglement and high-precision metrology. Phys. Rev. A.

[21] Scali S, Franzosi R (2019). Entanglement estimation in non-optimal qubit states. Ann. Phys..

[22] Ollivier H, Zurek WH (2001). Quantum discord: A measure of the quantumness of correlations. Phys. Rev. Lett..

[23] Cocchiarella D (2020). Entanglement distance for arbitrary $$m$$-qudit hybrid systems. Phys. Rev. A.

[24] Horodecki R, Horodecki M (1996). Information-theoretic aspects of inseparability of mixed states. Phys. Rev. A.

[25] Aaronson B, Lo Franco R, Adesso G (2013). Comparative investigation of the freezing phenomena for quantum correlations under nondissipative decoherence. Phys. Rev. A.

[26] Peres A (1996). Separability criterion for density matrices. Phys. Rev. Lett..

[27] Horodecki M, Horodecki P, Horodecki R (1996). Separability of mixed states: Necessary and sufficient conditions. Phys. Lett. A.

[28] Werner RF (1989). Quantum states with Einstein–Podolsky–Rosen correlations admitting a hidden-variable model. Phys. Rev. A.

[29] Pittenger AO, Rubin MH (2000). Note on separability of the Werner states in arbitrary dimensions1this work was supported in part by the national security agency 1. Opt. Commun..

[30] Dür W, Cirac JI (2000). Classification of multiqubit mixed states: Separability and distillability properties. Phys. Rev. A.

[31] Eltschka C, Siewert J (2012). Entanglement of three-qubit greenberger-horne-zeilinger-symmetric states. Phys. Rev. Lett..

[32] Schack R, Caves CM (2000). Explicit product ensembles for separable quantum states. J. Mod. Opt..

[33] Gühne O, Seevinck M (2010). Separability criteria for genuine multiparticle entanglement. New J. Phys..

